# Bayesian estimation of partial population continuity using ancient DNA and spatially explicit simulations

**DOI:** 10.1111/eva.12655

**Published:** 2018-07-03

**Authors:** Nuno Miguel Silva, Jeremy Rio, Susanne Kreutzer, Christina Papageorgopoulou, Mathias Currat

**Affiliations:** ^1^ AGP Lab Department of Genetics & Evolution – Anthropology Unit University of Geneva Geneva Switzerland; ^2^ Palaeogenetics Group Institute of Anthropology Johannes Gutenberg University Mainz Germany; ^3^ Laboratory of Physical Anthropology Department of History & Ethnology Democritus University of Thrace Komotini Greece; ^4^ Institute of Genetics and Genomics in Geneva (IGE3) Geneva Switzerland

**Keywords:** ancient DNA, genomewide autosomal data, mtDNA, Neolithic transition in Europe, partial population continuity, population genetics, serial coalescent, spatial explicit simulations

## Abstract

The retrieval of ancient DNA from osteological material provides direct evidence of human genetic diversity in the past. Ancient DNA samples are often used to investigate whether there was population continuity in the settlement history of an area. Methods based on the serial coalescent algorithm have been developed to test whether the population continuity hypothesis can be statistically rejected by analysing DNA samples from the same region but of different ages. Rejection of this hypothesis is indicative of a large genetic shift, possibly due to immigration occurring between two sampling times. However, this approach is only able to reject a model of full continuity model (a total absence of genetic input from outside), but admixture between local and immigrant populations may lead to partial continuity. We have recently developed a method to test for population continuity that explicitly considers the spatial and temporal dynamics of populations. Here, we extended this approach to estimate the proportion of genetic continuity between two populations, using ancient genetic samples. We applied our original approach to the question of the Neolithic transition in Central Europe. Our results confirmed the rejection of full continuity, but our approach represents an important step forward by estimating the relative contribution of immigrant farmers and of local hunter‐gatherers to the final Central European Neolithic genetic pool. Furthermore, we show that a substantial proportion of genes brought by the farmers in this region were assimilated from other hunter‐gatherer populations along the way from Anatolia, which was not detectable by previous continuity tests. Our approach is also able to jointly estimate demographic parameters, as we show here by finding both low density and low migration rate for pre‐Neolithic hunter‐gatherers. It provides a useful tool for the analysis of the numerous ancient DNA data sets that are currently being produced for many different species.

## 1 INTRODUCTION

Genetic diversity within species reflects past demographic changes and migrations. While genetic data from contemporary humans have long been the sole source of molecular information used to draw inferences on the evolution and peopling history of our ancestors (e.g., Cavalli‐Sforza, Menozzi, & Piazza, 1994), direct evidences from the past can now be recovered by sequencing ancient DNA (aDNA) from different time periods and geographical regions (e.g., Brandt et al., 2013; Gamba et al., 2012; Haak et al., 2005, 2010; Unterländer et al., 2017). Although full ancient genomes are now published with various levels of coverage (e.g., Bollongino et al., 2013; Broushaki et al., 2016; Hofmanová et al., 2016; Lazaridis et al., 2014), data sets belonging to the same prehistoric “population,” either geographically or culturally, have been published primarily for mitochondrial HVS1, such as for the Late Upper Paleolithic and Neolithic era in Central and Western Europe (Bramanti et al., 2009; Gamba et al., 2012). Those data have been used to address questions regarding whether there is continuity in the peopling history of a given area, thereafter called “population continuity,” in contrast to a full or partial population replacement due to the arrival of immigrants (Bramanti et al., 2009). Measuring the amount of genetic differentiation between two samples from different time periods, but located in the same geographical area (thereafter denominated “serial samples”), may help to differentiate these two hypotheses. Indeed, if the immigrant population arriving between the two sampling times is sufficiently large in number and genetically differentiated from the local population, a genetic shift might result. Hence, evidence of genetic discontinuity between serial samples indicates an absence of population continuity, whereas the reverse is not necessarily true. Indeed, if immigration were not sufficiently important in number or coming from a population that is not sufficiently genetically differentiated, then it could leave no distinguishable genetic trace.

A test of population continuity using serial samples, based on coalescent simulators (Anderson, Ramakrishnan, Chan, & Hadly, 2005; Excoffier & Foll, 2011), has been developed (Bramanti et al., 2009; Haak et al., 2010; Malmström et al., 2009). This test consists of simulating genetic data comparable to the real data under the null hypothesis of population continuity. The genetic differentiation between the two serial samples is estimated using the statistics *F*_st_ both for the observed and the simulated data. Coalescent simulations account for the stochasticity of the genealogical reconstruction, and thus, many simulations are performed and yield a distribution of the simulated *F*_st_ under the assumption of population continuity. Then, the proportion of simulated *F*_st_ that are greater than that observed is computed, and if it is smaller than 5%, the genetic shift is considered to be large enough to reject the population continuity hypothesis. This test has been recently improved by including two fundamental elements that were not considered in previous approaches: population subdivision and migration (Silva, Rio, & Currat, 2017). Under these continuity model assumptions, the genetic difference between the serial samples is due to sampling, genetic drift and ongoing gene flow with neighbours (Silva et al., 2017). A rejection of the continuity hypothesis may thus indicate that genetic input due to immigration occurred during the period separating the two sampling times and was sufficient to create a detectable shift in allele frequency. A substantial demographic replacement is generally invoked to explain the rejection of the test. For instance, a rejection of population continuity between Palaeolithic or Mesolithic hunter‐gatherers (PHG) and Neolithic farmers (NFA) from the same area could be interpreted as a partial or full demic replacement of the PHG by the NFA arriving from another region.

Ancient mitochondrial samples from different areas in Europe have been studied independently, and most analyses have revealed an absence of regional population continuity over time, either from prehistoric times until today, or between two ancient periods (Deguilloux & Mendisco, 2013). However, the coalescent‐based methods used to date are able to provide only a dichotomous answer (i.e., reject or accept full population continuity) but are unable to differentiate among different amount of genetic contribution (or replacement) from one period to the other. The distinction between full and partial population continuity is important because admixture between the local and immigrant populations is likely to occur, except in extreme cases of extermination due to warfare or disease. As a consequence, even if the arrival of immigrants disturbs the signal of genetic continuity, a full replacement of the local population does not necessarily occur.

A new spatially explicit simulation framework has been recently developed to investigate various evolutionary scenarios using aDNA (Silva et al., 2017). Based on this framework, we extended here the population continuity test by estimating partial continuity with serial samples. In contrast to previous studies, which have been limited to the complete rejection of population continuity (Bramanti et al., 2009; Haak et al., 2010; Silva et al., 2017), to the comparison between continuity or discontinuity models (Belle, Ramakrishnan, Mountain, & Barbujani, 2006; Ghirotto et al., 2013), or to the estimation of the maximum genetic contribution (Sjödin, Skoglund, & Jakobsson, 2014), our approach goes one step further by estimating the partial genetic continuity between two periods of time. We used our modelling framework to simulate the contact between two populations and to parameterize the amount of gene flow between them, using an assimilation rate *γ*. This rate of assimilation can be estimated to best fit the observed data and then translated into the relative genetic contribution of the two interacting populations to a descendant population. Our approach is also able to consider temporal variance and geographic variance, which are commonly found within aDNA population samples (Silva et al., 2017). Indeed, owing to the scarcity of prehistoric aDNA samples, lineages grouped in the same population sample, on a cultural and/or geographical basis, may be distant both temporally and geographically (e.g., Bramanti et al., 2009). Moreover, our framework allows for the simulation of different kinds of genetic markers, from full DNA sequences to SNPs or STRs, for haploid or diploid data.

The Neolithic transition involved a shift from subsistence practices based on hunting and gathering to an era predominantly characterized by farming activities (Ammerman & Cavalli‐Sforza, 1984; Childe, 1925; Price, 2000; Whittle & Cummings, 2007). In Europe, this transition occurred during the period from approximately 9,000 BP to 5,000 BP, spreading from the south‐east of the continent towards the north‐west (Ammerman & Cavalli‐Sforza, 1984; Bellwood, 2004; Özdoğan, 2010). Patterns of discontinuity between hunter‐gatherers and NFA were identified in different areas using mitochondrial data (Bramanti, 2008; Brandt et al., 2013; Chandler, Sykes, & Zilhão, 2003; Gamba et al., 2012; Haak et al., 2005, 2010; Hervella et al., 2012; Lacan et al., 2011; Lazaridis et al., 2014; Lee et al., 2012), Y chromosome data (Haak et al., 2010; Lazaridis et al., 2014; Szécsényi‐Nagy et al., 2015) and genomewide data (Lazaridis et al., 2014; Olalde et al., 2014; Raghavan et al., 2014; Sánchez‐Quinto et al., 2012; Skoglund et al., 2012). In particular, population replacement appears to have been important during the early phase of the Neolithic transition in Central Europe, with an arrival of immigrant farmers from the Aegean region (western Anatolia and eastern Greece, Hofmanová et al., 2016). Nevertheless, the influence of the hunter‐gatherers may have been stronger in later phases (Ehler & Vancata, 2009; Haak et al., 2015; Hofmanová et al., 2016).

Here, we applied our new spatially explicit simulation approach to obtain a more precise picture of the individual contributions of PHG and NFA to the final Neolithic population of Central Europe rather than simply rejecting full population continuity. We used both a mitochondrial data set and a genomewide autosomal data set of ancient PHG and NFA samples to estimate partial population continuity in this area during the Neolithic transition.

## 2 MATERIALS AND METHODS

### 2.1 Spatially explicit simulation of the Neolithic transition and aDNA

A serial version of the program SPLATCHE2 (Ray, Currat, Foll, & Excoffier, 2010) allowing for the sampling of lineages at different time points was used, thus making it possible to reconstruct the coalescent tree for genetic samples of different ages (Silva et al., 2017). This serial version is available online at http://www.splatche.com. This framework runs in two consecutive simulation steps, demographic then genetic (Currat, Ray, & Excoffier, 2004). The first step comprises the simulation of population densities and migration in a grid of demes exchanging migrants in a stepping‐stone fashion (Kimura, 1953). In the second step, coalescent reconstructions are performed to generate genetic diversity in samples of various ages and locations that are drawn from the simulated populations. The genealogy of simulated lineages is reconstructed in a manner conditional on the density and migration values calculated during the first step. The molecular diversity of those lineages is then simulated by distributing mutations on the coalescent tree. See the original description of SPLATCHE2 for more details on the algorithms (Ray et al., 2010).

#### 2.1.1 Virtual map of Europe

**Figure 1:**
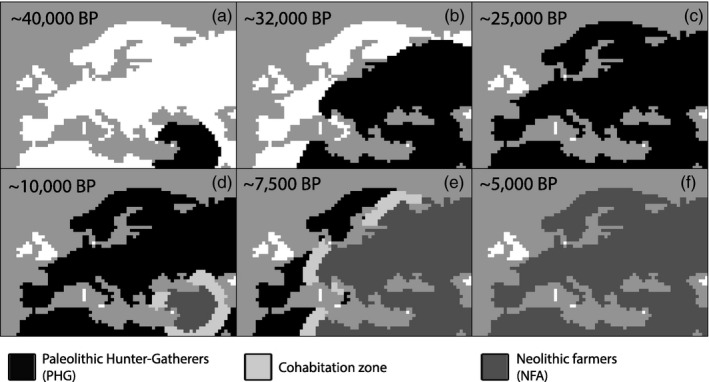
Temporal snapshots of the spatially explicit simulation framework used to estimate partial population continuity between pre‐Neolithic hunter‐gatherers (PHG) and Neolithic farmers (NFA) in Central Europe. Two successive population expansions are simulated in a digital map representing Europe divided into cells of 100 km × 100 km. Grey cells represent water; white cells empty area; black cells PHG only; dark grey NFA only; and light grey cohabitation zone with both PHG and NFA

We adapted the methodological framework developed by Currat and Excoffier (2005) to simulate the expansion of modern humans in Europe starting at approximately 40,000 years ago, followed by the Neolithic transition starting at approximately 10,000 years ago, in a digital map of Europe divided into cells of 100 km × 100 km (Figure 1). A smaller resolution (i.e., 50 km × 50 km) would not allow reproducing the rapid Neolithic spread from the Aegean area to Central Europe in <1,000 years (~40 generations) because at maximum one cell can be colonized per generation with our dispersal framework (short‐scale migrations). Note that we did only simulate continental populations. Despite our focus on the genetic influence of the Neolithic transition in Central Europe, we decided to simulate the entire continent for the past 40,000 years, because this model has already proven to be valuable (Arenas, François, Currat, Ray, & Excoffier, 2013; Currat & Excoffier, 2005; Silva et al., 2017) and to avoid any possible edge effects (Ray, Currat, & Excoffier, 2003). Most important, this modelling framework allowed us to consider the effects of surfing mutations (Klopfstein, Currat, & Excoffier, 2006), which could have important implications in the Neolithic transition (Currat & Excoffier, 2005; François et al., 2010). Although we simulated the entire European continent, the parameters were adjusted to fit the archaeological context of Central Europe because it constituted the zone of interest in this study. All density and carrying capacity values are given in the effective haploid size to simulate mitochondrial diversity and were multiplied by four when simulating autosomal diversity.

We used the two population layers mode of SPLATCHE2 and a generation time of 25 years. In brief, each cell contains two demes, each one representing a different population (PHG or NFA). We can therefore represent the whole grid of cells as two superimposed layers of demes, each layer representing a population (PHG or NFA) with its own migratory and demographic characteristics. The gene flow between demes belonging to the same geographic cell is regulated by the parameter *γ*, which is called thereafter the assimilation rate.

#### 2.1.2 PHG layer

The first layer represents the expansion of a PHG population of 100 individuals starting 1,600 generations ago (~40,000 years) from the Middle East (Figure 1a). For the mitochondrial data set, each PHG deme has a carrying capacity (*K*_PHG_) of 150 corresponding to 0.06 individuals/km^2^ (Alroy, 2001; Steele, Adams, & Sluckin, 1998). Indeed, 0.06 × 100 km × 100 km = 600 census individuals that correspond to 300 census females and to 150 effective females using a ratio effective vs census size equal to 1/2. The migration rate (*m*_PHG_) and growth rate (*r*_PHG_) were calibrated to 0.15 and 0.2 (Table 2), respectively, using 500 generations as the time of colonization of Europe by *Homo sapiens*, based on the simulation framework designed by Currat and Excoffier (2005). The migration rate corresponds to the proportion of individuals in each deme emigrating at each generation towards the demes belonging to the same population located in the neighbouring cells.

#### 2.1.3 NFA layer

The second layer represents the Neolithic expansion starting from Near East (Figure 1d) 400 generations before the present (~10,000 years). The source NFA population is made up of 100 individuals. Using a larger initial population size does not affect the results (Supporting Information Figure S1). Each NFA deme has a carrying capacity (*K*_NFA_) of 1,000 individuals which corresponds to the density estimated for the LBK (*LinearBandKeramic*), equal to ~0.6 individuals/km^2^ (Zimmermann, Hilpert, & Wendt, 2009). For the Neolithic layer, the migration rate (*m*_NFA_) and growth rate (*r*_NFA_) were calibrated to fit the dates of the early Neolithic samples under study. A minimum migration rate *m *=* *0.4 and a growth rate *r *=* *0.53 were estimated, corresponding to the speed of the spread of farmers in Europe, which was equal to 1.13 km/year, in accordance with an estimation based on archaeological data (Pinhasi, Fort, & Ammerman, 2005). In our model, NFA gradually replace PHG, owing to a competitive advantage driven by a higher carrying capacity.

#### 2.1.4 Assimilation rate

The gene flow *A* between the two layers depends on the parameter *γ*, the assimilation rate, following Currat and Excoffier (2005):

$$A=\frac{\gamma (2N_{\mathrm{NFA}}N_{\mathrm{PHG}})}{{\left(N_{\mathrm{NFA}}+N_{\mathrm{PHG}}\right)}^{2}}$$

where *N*_PHG_ and *N*_NFA_ are the number of PHG and NFA in the cell, respectively. *γ* controls the gene flow from PHG to NFA and represents the proportion of hunter‐gatherers adopting farming after contact with the farmers or the proportion of offspring with one parent PHG and one parent NFA. The assumption is made that all offspring with mix ascendance belong to the farming population, so assimilation occurs only from the PHG to the NFA and not in the reverse direction. A *γ* equal to its minimum, 0.0, indicates absence of gene flow between both layers because no assimilation of PHG into the NFA population occurs. A *γ* equal to its maximum, 1.0, indicates that PHG mix as much with NFA than with PHG, and it results to a full assimilation process at the continental scale, the spread of initial NFA genes being restricted to the source area (Currat & Excoffier, 2005).

Note that the migration rate *m* represents gene flow between demes belonging to the same population (PHG or NFA) but located in adjacent cells, while the assimilation rate *γ* represents gene flow between different populations (PHG and NFA) located in the same cell.

### 2.2 Data set analysed

**Figure 2:**
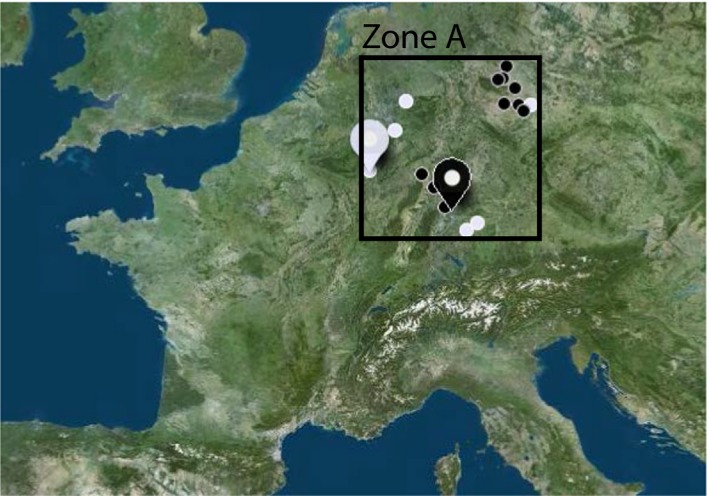
Geographical locations of the mitochondrial and genomewide autosomal data used in this study. Hunter‐gatherers (white) and farmers (black) samples are shown on the map as small and large circles for the mtDNA and autosomal data, respectively. The area corresponding to the zone A in Figure 3 is indicated

We simulated genetic samples identical to real data in terms of lineage number, geography and chronology (Table 1 and Figure 2).

**Table 1:** Characteristics of the ancient mitochondrial and genomewide autosomal (*) samples used in the analyses, including temporal and geographic information

Group	Pop.	Sample count	Site	Geographic region	Sample age (cal BCE)	Archaeological context	Latitude	Longitude	Individuals per site	References
Hunter gatherer central Europe	PHG	11/1*	Hohler Fels	Germany	13,400	Paleolithic	48.38	9.76	1	Bramanti et al. (2009)
			Bad Dürrenberg	Germany	6,990–6,706	Mesolithic	51.30	12.07	1	Bramanti et al. (2009)
			Hohlenstein‐Stadel	Germany	6,743	Mesolithic	48.55	10.17	2	Bramanti et al. (2009)
			Oberkassel	Germany	11,641–11,373	Paleolithic	50.71	7.17	1	Fu et al. (2013)
			Blätterhöhle	Germany	9,210–8,638	Mesolithic	51.36	7.55	5	Bollongino et al. (2013)
			Loschbour	Luxembourg	6,147–6,047	Mesolithic	49.77	6.24	1 + 1*	Lazaridis et al. (2014)
Early Neolithic central Europe (LBK)	NFA	99/1*	Derenburg	Germany	5,500–4,775	LBK	51.87	10.91	20	Haak et al. (2005, 2010), Brotherton et al. (2013)
			Halberstadt‐Sonntagsfeld	Germany	5,500–4,775	LBK	51.90	11.06	31	Haak et al. (2005), Brandt et al. (2013)
			Karsdorf	Germany	5,500–4,775	LBK	51.28	11.65	23	Brandt et al. (2013), Brotherton et al. (2013)
			Naumburg	Germany	5,500–4,775	LBK	51.15	11.81	4	Brandt et al. (2013)
			Oberwiederstedt 1, Unterwiederstedt	Germany	5,500–4,775	LBK	51.67	11.53	8	Haak et al. (2005), Brandt et al. (2013)
			Eilsleben	Germany	5,000	LBK	52.15	11.22	1	Haak et al. (2005)
			Schwetzingen	Germany	5,500–4,775	LBK	49.38	8.57	4	Haak et al. (2005)
			Vaihingen	Germany	5,500–4,775	LBK	48.93	8.96	1	Haak et al. (2005)
			Seehausen	Germany	5,500–4,775	LBK	51.33	11.13	1	Haak et al. (2005)
			Flomborn	Germany	5,500–4,775	LBK	49.69	8.15	6	Haak et al. (2005)
			Stuttgart	Germany	5,500–4,800	LBK	48.86	9.22	1*	Lazaridis et al. (2014)

*Note*

NFA, Neolithic farmers; PHG, pre‐Neolithic hunter‐gatherers.

#### 2.2.1 Mitochondrial data set

The mitochondrial data set was constructed by collecting from the literature sequences of hunter‐gatherers and early farmers from Central Europe, which were sufficiently close chronologically and geographically to be grouped together to represent a population sample. The data set constituted 99 farmers and 11 hunter‐gatherer HV1 sequences of 344 bp length (five additional positions were not included due to missing data in the entire data set). We used a mutation rate of 7.5 × 10^−6^ mutations per generation per site (Bramanti et al., 2009) to simulate DNA sequences molecular diversity.

#### 2.2.2 Genomewide autosomal data set

For the autosomal analysis, we chose two ancient genomes with sufficient coverage (one PHG and one NFA), as close as possible to the area where the mitochondrial data set had been taken: A hunter‐gatherer genome from the end of the Paleolithic (Loschbour, 8kya, Lazaridis et al., 2014) and a farmer genome from the beginning of the Neolithic (Stuttgart, 7kya, Lazaridis et al., 2014).

### 2.3 Generation of autosomal SNPs

To reproduce genomewide autosomal data with characteristics similar to those of the real data, we performed a harmonization step and a calibration step. To do so, we simulated a reference panel of seven modern genomes in addition to the two ancient ones. The number and location of the reference genomes corresponded to the real population samples published by Lazaridis et al. (2014), which were geographically the closest to the ancient genome locations (“French South”). Ten thousand (10,000) independent SNPs (single nucleotide polymorphisms) were simulated for each autosomal simulation. A series of preliminary simulations indicated that this was the minimum value to get robust estimate and that the exact location of modern genomes used as reference did not affect the results of the estimation (not shown).

#### 2.3.1 SNP harmonization

Each SNP generated by SPLATCHE2 was variable among the nine simulated genomes (i.e., there was at least one derived allele). Each real SNP was variable among all genomes belonging to the whole reference database, but not always among the nine genomes analysed. In consequence, we removed from the analysis all positions that were homozygous for the same allele in all nine real genomes (modern and ancient), thus resulting in 207,591 SNP positions overlapping among all nine genomes. Then, allele 1 in SPLATCHE2 was the derived allele, whereas in the real data set, that allele did not have the same significance: It was the alternative allele to the reference. Therefore, we performed a folding procedure in which 0 became the most frequent allele at each position, both in the real and simulated data. This folding had no consequence on the three statistics computed but affected only the computation of the site frequency spectrum (SFS), as shown below.

#### 2.3.2 SNP calibration

Rare alleles were overrepresented in our simulated data set compared with the real data set. To remove this bias, we performed a calibration procedure in two steps. First, we set the minimum frequency of the derived allele in SPLATCHE2 to 0.12. A series of preliminary simulations revealed that this was the most optimized value to accelerate the computational time. Second, we computed the SFS of the real seven modern genomes, and for each simulation, a subset of SNPs was created from the full set of simulated SNPs to reproduce an SFS for modern data that was similar to the real SFS. A lower number of real modern genomes would result in a lower calibration accuracy, while a higher number would be time‐consuming.

At last, a comparison between observed and simulated data was performed based on statistics computed on only the ancient genomes. The averages computed on the full set of 207,591 overlapping SNP were used as observed values, to which the average simulated statistical results were compared (see below).

### 2.4 Estimation of parameters using ABC

To estimate the assimilation rate *γ* between PHG and NFA during the Neolithic transition in Central Europe, we applied the approximate Bayesian computation (ABC, Beaumont, Zhang, & Balding, 2002) approach using the ABCtoolbox package (Wegmann, Leuenberger, Neuenschwander, & Excoffier, 2010). To address the uncertainty of the six demographic parameters (migration rate, growth rate and carrying capacity for both layers), we defined prior distributions based on the values estimated from the literature as described above (Table 2). The parameter *γ* was sampled from a uniform distribution between 0.0 and 0.15 for the mitochondrial data set and between 0.0 and 0.2 for the autosomal data set, because statistical variation mostly occurs for low values of *γ* (Supporting Information Figure S2). Moreover, a previous round of simulations showed that values equal or higher than 0.2 do not fit the observed statistics (Supporting Information Figure S3) and that prior too wide towards large values tend to bias the estimation of *γ* (Supporting Information Figures S4C and S5C).

**Table 2:** Description and characteristics of the prior distributions for all the model parameters used for the simulations

Parameters	Description	Distribution	Min	Max
*γ*	Assimilation rate between PHG and NFA	Uniform	0^a^	0.15^a^
			0^b^	0.2^b^
*r*_PHG_	Growth rate in PHG	Uniform	0.2	0.4
*m*_PHG_	Migration rate in PHG	Uniform	0.15	0.3
*K*_PHG_	Carrying capacity in PHG	Uniform	50^a^	250^a^
			200^b^	1,000^b^
*r*_NFA_	Growth rate in NFA	Uniform	0.53	0.7
*m*_NFA_	Migration rate in NFA	Uniform	0.4	0.8
*K*_NFA_	Carrying capacity in NFA	Uniform	500^a^	2,500^a^
			2,000^b^	10,000^b^

*Notes*

NFA, Neolithic farmers; PHG, pre‐Neolithic hunter‐gatherers. ^a^Specific to mitochondrial data. ^b^Specific to autosomal data.

Many simulations were performed with distinct combinations of parameters drawn from prior distributions: 320,000 for the mitochondrial analysis and 60,000 for the autosomal analysis (due to a much longer computational time). For each simulation, the *F*_st_ between the two samples (PHG and NFA) in Central Europe is computed with the program Arlequin (Excoffier & Lischer, 2010) as $F_{\mathrm{st}}=\sigma_{a}^{2}/\sigma_{T}^{2}$ where $\sigma_{a}^{2}$ is the covariance component due to differences among populations and $\sigma_{T}^{2}$ the total molecular variance. We also computed with Arlequin the heterozygosity, *H* (gene diversity for mtDNA), within each sample (PHG and NFA). The three statistics were used to estimate *γ* and its 50 and 90 highest posterior density intervals (HDI 50 and HDI 90). The six other parameters were also estimated. A fraction *δ* equal to 1% of the simulations that produced the simulated statistics that were the closest to the observed ones were retained (Csillery, François, & Blum, 2012) and used to estimate the most plausible parameter values. Based on this fraction *δ* of the retained simulations, we estimated each parameter independently using the ABC‐GLM method implemented in the ABCtoolbox (Wegmann et al., 2010). Note that some combinations of parameters did not allow sampling in the PHG layer due to the too rapid disappearance of PHG consecutive to the spread of NFA. This is mostly the case when *m*_NFA_ and *γ* are both high when simulating autosomal data. These unlikely parameter combinations are nevertheless considered in the ABC estimation by the fact that they never appear in the retained simulations.

### 2.5 Cross‐validation procedure

To evaluate the ability of our model to reproduce the observed data, we computed the marginal density and Tukey depth *p*‐values, an option available in the ABCtoolbox. Low *p*‐values indicate a poor model fit to the observed data with all the statistics considered. We used the same fraction *δ* = 1% of simulations to compute the marginal density and Tukey depth.

To evaluate the accuracy of our estimation of *γ*, we performed a posterior predictive check that consisted of visually assessing whether each observed statistic fell within the distribution of statistics simulated under the best set of parameters. The distribution of each statistic was calculated for 1,000 simulations using the estimated parameters as input.

Furthermore, we also generated 1,000 pseudo‐observed simulations (pods) that were used to evaluate the estimation of the parameters using the random validation mode of ABCtoolbox. These pods were generated by drawing parameters in their prior distributions (Table 2). We estimated the parameters ($\hat{\theta }$) for each of those pods using the same methodology as for the real data, the only difference being that the parameter values used to generate the data are known (*θ*). We computed five different indices to evaluate the precision of our estimation: the relative bias $=1/n\sum_{i=1}^{n}({\hat{\theta }}_{i}-\theta )/\theta$ and the relative root mean square error $=1/\theta \sqrt{1/n\sum_{i=1}^{n}({\hat{\theta }}_{i}{-\theta )}^{2}}$, both being relative so a value of 1 means 100% of the “true” value *θ*, where *n* is the number of pods. If the relative bias is positive or negative then the “true” value is overestimated or underestimated, respectively. To further assess the quality of the posterior distributions, we computed their 50% and 90% coverage, defined as the proportion of simulations in which the “true” value *θ* lies within the 50% (respectively, 90%) HDI around the estimate $\hat{\theta }$. To obtain information on the absolute precision of the estimator, we also computed the Factor 2 as the proportion of estimated values $\hat{\theta }$ lying in an interval bounded by 50% and 200% of the “true” value *θ*. (Neueunschwander et al., 2008).

Then, we also checked for potential biases in the posterior distribution of θ by computing with the ABCtoolbox the posterior cumulative probabilities corresponding to the true parameter values. If the posterior distribution is unbiased, then the posterior cumulative probability of the true parameter value is expected to be equally distributed over the 1,000 pods. Deviation from the uniform distribution was detected with the Kolmogorov–Smirnov test (Wegmann, Leuenberger, & Excoffier, 2009).

### 2.6 Computation of genetic contribution

**Figure 3:**
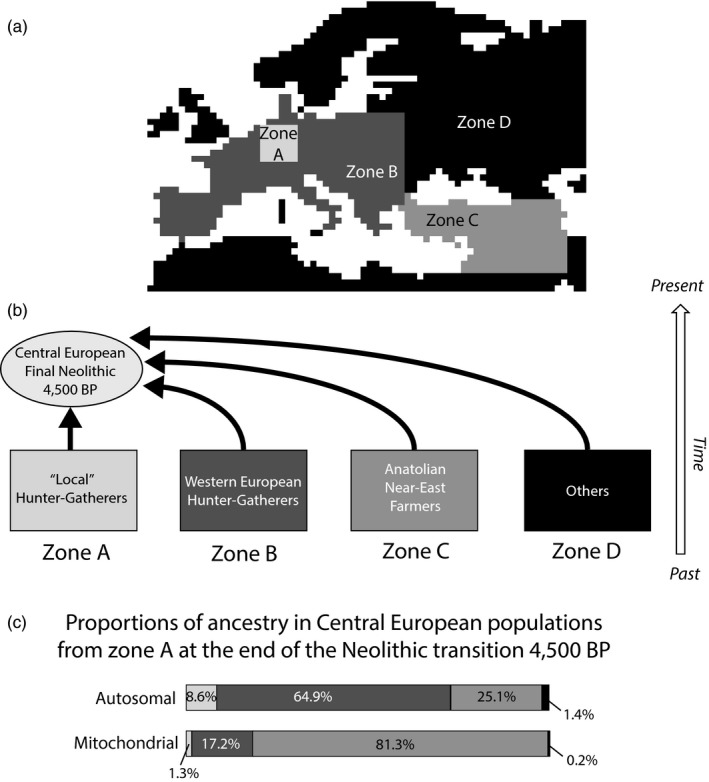
(a) Different zones defined for computing proportions of ancestry in Central Europeans 4,500 BP. (b) Schematic representation of various population contributions. (c) Mean proportions of ancestry from the various pre‐Neolithic hunter‐gatherers (PHG) zones (A+B+C+D) in Central European populations from zone A at the end of the Neolithic transition 4,500 BP, computed for autosomal and mitochondrial markers

We translated the assimilation rate values *γ* into the genetic contribution of PHG to the final Neolithic population, by following the approach of Currat and Excoffier (2005). For each simulation, we draw 25 samples of 50 genes regularly spread over Central Europe in the NFA layer (zone A in Figure 3) ~4,500 BP, a date approximately corresponding to the final Neolithic phase in this area (Whittle & Cummings, 2007). We traced the sampled lineages back in time through the coalescent reconstruction and located where they entered the NFA layer from the PHG layer. Is it in the local area where they have been sampled (zone A); somewhere else in Western Europe (zone B); in Anatolia (zone C); or elsewhere in the map (zone D)? We thus computed the proportion of NFA genes from zone A at the end of the Neolithic whose lineages go back to each of those different areas of the PHG layer (Zones A, B, C and D). Note that Zone A has been designed as the minimum rectangle area encompassing all the sampling locations. We computed these contributions for the 1% retained simulations for both the autosomal and the mitochondrial estimations.

## 3 RESULTS

We simulated the arrival of the early NFA in Central Europe and their interactions with local hunter‐gatherers (PHG) and the subsequent replacement of PHG because of a competitive advantage for NFA. During the cohabitation period, PHG may contribute to the farming community at various intensities, as regulated by the assimilation parameter *γ*. The maximum assimilation of PHG (*γ* = 1.0) represents an acculturation process (knowledge transfer from NFA to PHG), whereas the minimum assimilation (*γ* = 0.0) represents a full genetic replacement of PHG by NFA.

**Figure 4:**
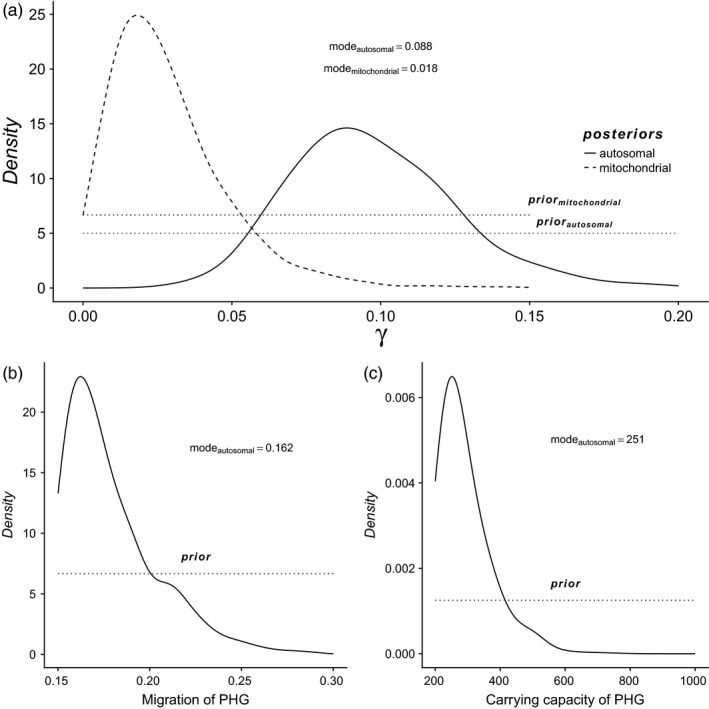
Prior (dotted) and posterior distributions of the estimated parameter for mtDNA (dashed) and genomewide autosomal data (solid). (a) *γ*, (b) *m*_PHG_, (c) *K*_PHG_. The mode of the posterior distribution is used as the point estimate

With both data sets, small values of *γ* were estimated in Central Europe: 0.018 (HDI90 = [0.0–0.055]) with the mitochondrial data set and 0.088 (HDI90 = [0.050–0.142]) with the genomewide autosomal data set (Figure 4a and Table 3). Those results indicate that approximately 2%–9% of the contacts between the PHG and NFA resulted in the adoption of farming by PHG or the birth of a child in the farming community with parents from both populations.

**Table 3:** Characteristics of the posterior distributions for the model's parameters for the mitochondrial and the autosomal data set, bold values show estimated parameters

Parameters	Data set	Posterior characteristics						
		Mode	Mean	Median	HDI50 lower	HDI50 upper	HDI90 lower	HDI90 upper
*γ*	Mitochondrial DNA	**0.018**	**0.029**	**0.025**	**0.009**	**0.031**	**0.000**	**0.055**
*r*_PHG_		0.31	0.30	0.30	0.29	0.39	0.22	0.39
*m*_PHG_		0.16	0.22	0.22	0.15	0.22	0.15	0.28
*K*_PHG_		115	148	147	59	154	57	232
*r*_NFA_		0.65	0.65	0.62	0.62	0.69	0.55	0.70
*m*_NFA_		0.75	0.61	0.61	0.60	0.79	0.44	0.79
*K*_NFA_		952	1,486	1,485	585	1,554	575	2,332
***γ***	Autosomal SNP	**0.088**	**0.097**	**0.095**	**0.072**	**0.110**	**0.050**	**0.142**
*r*_PHG_		0.27	0.29	0.29	0.21	0.29	0.21	0.38
***m***_**PHG**_		**0.16**	**0.18**	**0.17**	**0.15**	**0.18**	**0.15**	**0.22**
***K***_**PHG**_		**251**	**305**	**285**	**213**	**297**	**200**	**417**
*r*_NFA_		0.56	0.61	0.61	0.54	0.61	0.53	0.68
*m*_NFA_		0.49	0.56	0.55	0.44	0.58	0.40	0.70
*K*_NFA_		4,996	5,734	5,569	2,183	5,725	2,116	8,908

Even though estimates were obtained for the six other demographic parameters, we did not attempt to interpret them in detail, because the large associated HDI and flat posterior distribution indicated that the statistics used were not sufficiently informative regarding those parameters (Table 3). However, setting prior distributions for those variable parameters was useful to take their uncertainty into account when estimating the parameter of interest, *γ*. There are two notable exceptions, *m*_PHG_ and *K*_PHG_ (Figure 4b,c), which are estimated to the lower range of their respective prior distributions with a mode at 0.16 (HDI 90 = 0.15–0.22) and 251 (HDI 90 = 200–417), meaning *Nm* ~40, so ~40 genes (~20 individuals) exchanged between neighbouring demes on average per generation at demographic equilibrium. A *K*_PHG_ = 250 corresponds to 0.025 individuals/km^2^, a value about half of the one estimated for PHG (Alroy, 2001; Steele et al., 1998).

**Figure 5:**
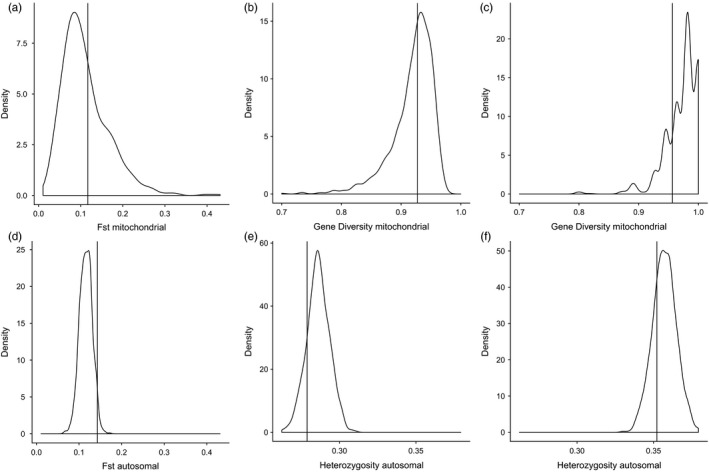
Postpredictive check plot of the *F*_st_, *H*_PHG_, and *H*_NFA_ statistics for the mitochondrial (a ‐ c) and autosomal analyses (d ‐ f). The distribution of each statistic is calculated for 1,000 simulations using the estimated parameters, and the observed values are indicated by vertical lines

The cross‐validation procedure indicated that our model robustly reproduced the observed statistics, as revealed by the marginal densities and Tukey depth *p*‐values of 0.90/0.85 (mitochondrial data) and 0.86/0.83 (autosomal data) and by the posterior predictive check (Figure 5). Moreover, it showed that the mode of the posterior distribution of *γ* is a better point estimate than the mean with a tendency to overestimate the true value by 11% for the mitochondrial data set (RMSE = 40%, Factor 2 = 82%, Coverage 50 = 52%, Coverage 90 = 92%) and by 7% for the autosomal data set (RMSE = 1%, Factor 2 = 93%, Coverage 50 = 56%, Coverage 90 = 89%). The autosomal estimation is thus more precise than the mitochondrial estimate, despite a lower number of individuals. A closer look to the variation of these indices through different range of *γ* shows that the precision of the estimation is maximum around 8%, then it tends to decrease with *γ* (Supporting Information Tables S1 and S2). The relative bias for the parameter *m*_PHG_ is 0.6% (RMSE = 22%, Factor 2 = 100%, Coverage 50 = 52%, Coverage 90 = 90%) and 5% for *K*_PHG_ (RMSE = 33%, Factor 2 = 91%, Coverage 50 = 50%, Coverage 90 = 91%).

Furthermore, the quantile distributions for *γ* did not reject uniformity at the 5% level for both the mitochondrial (Kolmogorov–Smirnov *p* value = 0.547) and autosomal data (Kolmogorov–Smirnov *p* value = 0.194), as expected for an unbiased estimation (Supporting Information Figure S4A,B). Among the other informative parameters with autosomal data, *m*_PHG_ does not reject uniformity (Kolmogorov–Smirnov *p* value = 0.712, Supporting Information Figure S4D) while *K*_PHG_ just rejects it (*p* value = 0.044, Supporting Information Figure S4E) due to a prior with too large values (Figure 4c), but we checked that using a smaller upper range for *K*_PHG_ does not affect the estimation (not shown).

When translating the assimilation rate values *γ* into genetic contributions, we found that Central European farmers (zone A in Figure 3 and Supporting Information Figure S6) at the end of the Neolithic transition (~4,500 BP) have 8.6% autosomal ancestry (1.3% mitochondrial ancestry) coming from the local Central European hunter‐gatherers (same zone A), 64.9% (17.2% for mt) coming from other western European hunter‐gatherers (zone B) and 1.4% (0.2% for mt) coming from other areas than Western Europe, Anatolia and the Near‐East (zone D). The remaining 25.1% ancestry (81.3% for mt) is coming from early farmers from Anatolia and the Near‐East (zone C).

Those results imply that in Central Europe at the end of the Neolithic (4,500 BP), more than 90% of the autosomal genetic pool resulted from the arrival of expanding farmer populations during earlier phases of the Neolithic. This number is even higher regarding the mitochondrial genetic pool (>98%). However, a majority of those incoming genes were already present in other western European hunter‐gatherer populations and were acquired by expanding farmers on their way from Anatolia to Central Europe. We computed that 73.5% of the autosomal and 18.5% of the mitochondrial genes of Central European farmer populations around 4,500 BP were already present in other hunter‐gatherer populations from Western Europe before the arrival of farming (Figure 3).

When looking at the spatial distribution of common ancestors of lineages sampled in Central Europe (coalescent events) for one single simulation (Supporting Information Figure S7A) and over 1,000 simulations (Supporting Information Figure S7B), it is visible that most coalescent events occurred along a south‐east to north‐west axis, corresponding to the direction of the two main population expansions (Paleolithic and Neolithic). It roughly shows the geographic origins of the ancestors of the people in Central Europe at the end of the Neolithic period.

## 4 DISCUSSION

By simulating two population layers in SPLATCHE2, one representing the PHG and the other representing the NFA, with various levels of assimilation from PHG to NFA, as regulated by the parameter *γ*, we were able to estimate the amount of genetic continuity during the Neolithic period, which was compatible with ancient mitochondrial and autosomal data in Central Europe. In accordance with the conclusion previously obtained with a nonspatial model (Bramanti et al., 2009) and a spatial model that does not account for partial contribution (Supporting Information Appendix S1), our new approach rejected full population continuity (<100% contribution of local PHG to the Neolithic community) based on both mtDNA and autosomal data. Note that both continuity tests, panmictic (Bramanti et al., 2009) and spatial (Silva et al., 2017), use *F*_st_ as a single statistics while our approach also makes use of statistics measuring intrapopulation diversity. Our approach could potentially be used with additional statistics depending on the kind of data analysed, but it needs genomes with sufficient coverage to have enough overlapping loci.

However, our approach does not yield only a dichotomous answer to the population continuity test (reject or accept full continuity); instead, it goes one step further by estimating the most probable amount of local genetic contribution. Both data sets were consistent with a low estimated *γ* (1.8% for mtDNA and 8.8% for autosomal data), meaning that about 2%–9% of the contacts between the PHG and NFA resulted in the adoption of the Neolithic way of life by the hunter‐gatherers. Independent of the accuracy of the point estimate, our results point to a larger genetic replacement with mitochondrial than with autosomal data. The difference between the two posterior distributions for *γ* may be potentially due to the differences between the two data sets analysed: 1/the exact location of samples (see Figure 2); 2/the sample sizes (2 genomes vs. 109 mitochondrial sequences); 3/a single uniparental locus versus multiple autosomal loci; 4/the kind of molecular markers (full DNA sequence vs. SNP). Note that our approach takes into account the difference in number of gene copies between the two data sets. Nevertheless, the difference between the mitochondrial and autosomal posterior distributions could also mean that fewer female versus male hunter‐gatherers were assimilated into the farming community, given that mitochondria are transmitted through the maternal line only. Indeed, more reproduction between NFA males and PHG females than the opposite is expected to lead to an estimation of *γ* higher for mitochondria than for autosomes (or the Y chromosome). This hypothesis needs to be investigated further with more comparable data; for instance, by applying our method to an ancient data set for which mitochondria, the Y chromosome and autosomes were typed in the same set of individuals, to avoid at maximum potential bias in the estimation due to the difference of data sets.

We translated the estimated assimilation rate into the respective contributions of the PHG and NFA groups to the genetic pool of the final Neolithic populations from Central Europe. We estimated that only 8.6% of the autosomal genetic pool has its ancestry in local Central European PHG populations (1.3% for mitochondria). However, among the 91.4% entering Central Europe during the Neolithic transition, nearly two of three traces their ancestry back to PHG located along the Neolithic route from Anatolia (~1/6 for mitochondria). It is due to the effect of continuous adoption of farming by PHG during the Neolithic spread from the Aegean area (Currat & Excoffier, 2005). In consequence, about 25% of the genetic pool at the end of the Neolithic transition in Central Europe traces its ancestry back to Anatolia or further East (83.8% for mitochondria), consistently with an arrival of Neolithic immigrants from south‐eastern Europe (Hofmanová et al., 2016; Mathieson et al., 2015; Omrak et al., 2016). The relatively low contribution of Central European hunter‐gatherers (between ~2% and ~9%) could be partly due to the fact that we analysed data from the earliest Neolithic phase and that local genetic contribution has possibly increased during the later Neolithic phases through the effects of continuous contact between the early farming communities and the surrounding forager populations (Lazaridis et al., 2014). The lower PHG genetic contribution estimated for mitochondrial data than for autosomal data can be explained both by the differences in the assimilation rate estimated (0.018 and 0.088, respectively) and by lower introgression of local genes in uniparental markers than in autosomal markers due to different effective sizes, as already noted in the case of admixture between Neanderthals and modern humans (Currat & Excoffier, 2011).

Our results are in general accordance with two distinct ancestry components that have previously been detected at the continental scale by Lazaridis et al. (2014): the “early European farmer” (EEF), which corresponds here to the NFA from Anatolia (zone C in Figure 3), and the “West European hunter‐gatherer” (WHG), which corresponds here to the PHG from zones A and B in Figure 3. In particular, the contribution of an Ancient North Eurasians (ANE) component is not included in our model as we did not consider potential post‐Neolithic immigration waves, which could have contributed to the modern European genetic pool, such as migration from the Pontic steppes associated with the Yamnaya culture (Haak et al., 2015). Without considering the ANE ancestry component, our estimate of the autosomal genetic contribution of Early farmers to the gene pool of Central European populations (25%) tends to be lower than the EEF ancestry estimated in most modern western European populations, but is of the same order than the estimations in modern Estonians and in the ancient Late Neolithic genome “Karsdorf” from Germany (Haak et al., 2015; Lazaridis et al., 2014). Note that the contribution of hunter‐gatherers to Neolithic communities appears to be variable in different regions of Europe (Brandt et al., 2013; Lazaridis et al., 2014; Skoglund et al., 2012), while we computed an average value for Central Europe. Moreover, we computed the ancestry of the two groups at the end of the Neolithic period while previous studies estimated it in modern times. At last, previous studies used molecular information to directly estimate admixture proportions, while we use molecular information to estimate the model parameters and, then, we computed the expected genetic contributions of both groups using the best parameters, without using molecular information during this second step. Model assumptions may thus influence the inferences on the relative genetic contribution of both groups. In particular, we made the assumption of a uniform expansion of NFA with constant and similar assimilation of PHG over the whole continent but spatio‐temporally heterogeneous environment, variable assimilation rate and long‐distance dispersal may have played an important role. The effects of those factors should be investigated in future studies.

The approach presented in this study is particularly well adapted to estimate partial continuity in different areas for which aDNA data are available. By modelling the entire continent, our approach considers the process of asymmetric introgression that occurs when a population expands into an occupied territory (Currat, Ruedi, Petit, & Excoffier, 2008). This asymmetrical gene flow results from the demographic and migratory dynamics of the interacting populations and leads to an increase in the frequency of some local genes in the expanding population, owing to mutation surfing (Klopfstein et al., 2006). It implies, on the one hand, that a small demographic contribution of hunter‐gatherers may lead to a significant genetic contribution to the final genetic pool and, on the other hand, that the genes of early NFA from south‐eastern Europe tend to dilute in the hunter‐gatherer genetic pool, as the population expands towards the north‐west (Currat & Excoffier, 2005). The Supporting Information Figure S8 illustrates this effect, we performed 1,000 simulations with parameters drawn from the prior distributions of Table 2, and we plotted against the assimilation rate *γ* the genetic contribution of PHG to lineages sampled in the NFA layer after the Neolithic expansion. It explains why an assimilation rate as low as 2%‐9% per generation leads to a substantial genetic contribution of western European hunter‐gatherers to the final farming community (~73% for autosomal data and ~18% for mitochondrial data). Future studies incorporating additional genomes, more statistics or additional information (i.e., archaeology, environment) could improve the precision of the estimates.

## 5 CONCLUSION

In summary, our results support an important immigration in Central Europe during the Early Neolithic phase, in agreement with findings from previous studies (e.g., Bramanti et al., 2009; Haak et al., 2005; Hofmanová et al., 2016). However, they suggest that the genetic legacy of western European hunter‐gatherer may have been substantial due to their assimilation in farming communities in a repeated manner during the Neolithic spread from the Aegean area.

Our approach is appropriate for estimating partial genetic continuity between two ancient periods or between ancient and modern times, in taking into account spatial factors. It can provide an estimate of the relative genetic contribution of a pair of population sources to a descendant population, even with an ancient data set of relatively limited size and heterogeneous in space and time. Moreover, the method has the potential to study sex‐specific patterns by comparing uniparental and autosomal genetic markers.

The approach is quite versatile and allows for the study of many different evolutionary scenarios with ancient molecular data. It will thus be particularly useful in the future to analyse new aDNA data sets not only in humans but also in other organisms.

## CONFLICT OF INTEREST

None declared.

## AUTHORS’ CONTRIBUTIONS

NMS and JR carried out simulations and analyses. NMS, JR and MC conceived, designed and coordinated the study and interpreted the results. SK and CP compiled the data. All authors contributed to the writing of the manuscript and gave final approval for publication.
