# Supplementary material for: Bayesian estimation of partial population continuity using ancient DNA and spatially explicit simulations
Source: Evol Appl. 2018 Jul 3;11(9):1642–55. doi: 10.1111/eva.12655 (PMC6183456; doi:10.1111/eva.12655)

Figure 1. Temporal snapshots of the spatially explicit simulation framework used to estimate partial population continuity between pre-Neolithic hunter-gatherers (PHG) and Neolithic farmers (NFA) in Central Europe. Two successive population expansions are simulated in a digital map representing Europe divided in cells of 100 *km* *100 *km*. Grey cells represent water; white cells empty area; black cells PHG only; dark grey NFA only; and light grey cohabitation zone with both PHG and NFA.

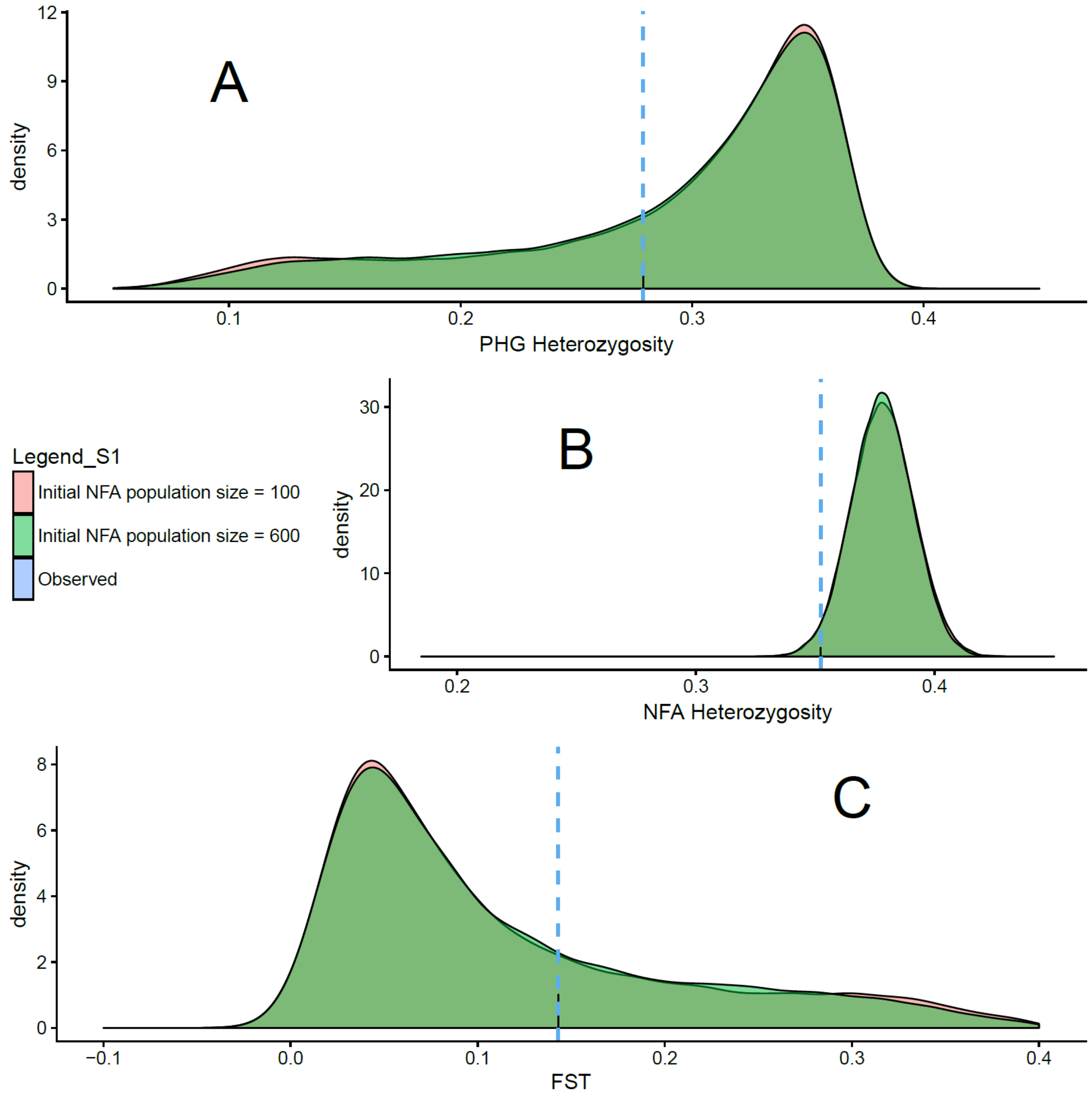

Supplement: Supplementary file 1 [file EVA-11-1642-s001.pdf]
