# Supplementary material for: Bayesian estimation of partial population continuity using ancient DNA and spatially explicit simulations
Source: Evol Appl. 2018 Jul 3;11(9):1642–55. doi: 10.1111/eva.12655 (PMC6183456; doi:10.1111/eva.12655)

Figure S2. Example of the evolution of the three autosomal statistics (*Fst* between PHG and NFA, Heterozygosity in NFA and in Heterozygosity PHG) for 4 different γ values (0, 0.05, 0.1 and 0.2) and for two values of K_{PHG} (300 and 600). The other parameters were fixed to: m_{PHG}=0.15, m_{NFA}=0.4, r_{PHG}=0.3, r_{NFA}=0.6, and K_{NFA} = 10x K_{PHG}.

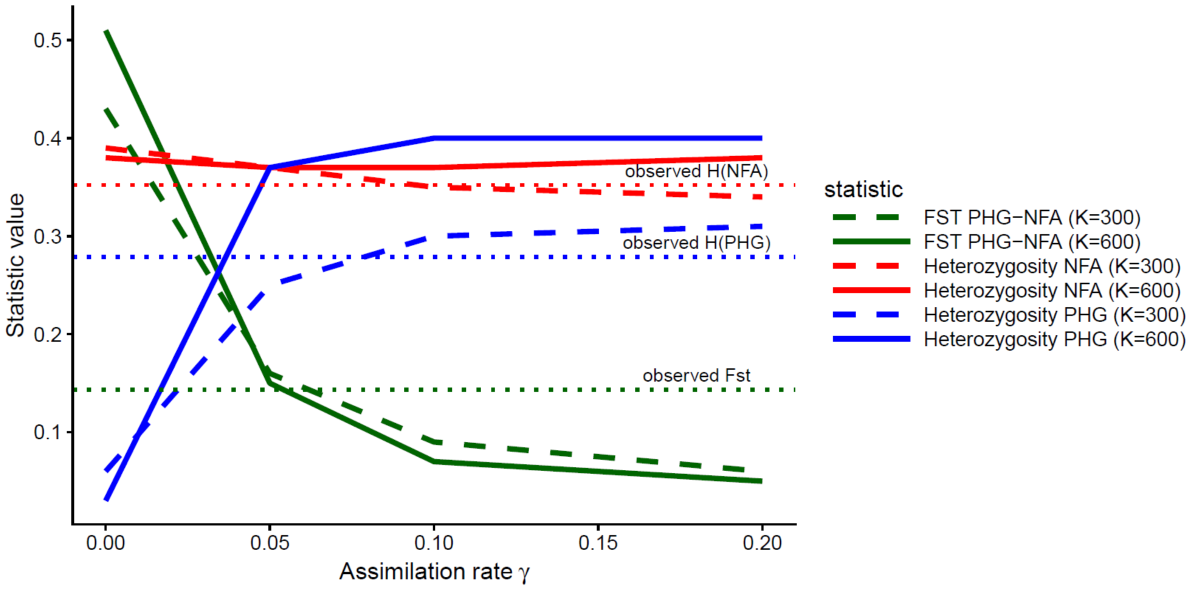

Supplement: Supplementary file 2 [file EVA-11-1642-s002.pdf]
