# Supplementary material for: Bayesian estimation of partial population continuity using ancient DNA and spatially explicit simulations
Source: Evol Appl. 2018 Jul 3;11(9):1642–55. doi: 10.1111/eva.12655 (PMC6183456; doi:10.1111/eva.12655)

Figure S3. Distribution of statistics computed for two series of 10,000 autosomal simulations with parameters taken from prior distributions of table 2. The two series differ only by the assimilation rate γ, which was either drawn from a prior distribution going from 0.0 to 0.15 (pink curve) or fixed to 0.2 (green curve). The blue line shows the observed statistics. A) Heterozygosity in PHG, B) Heterozygosity in NFA, C) *Fst* between PHG and NFA.

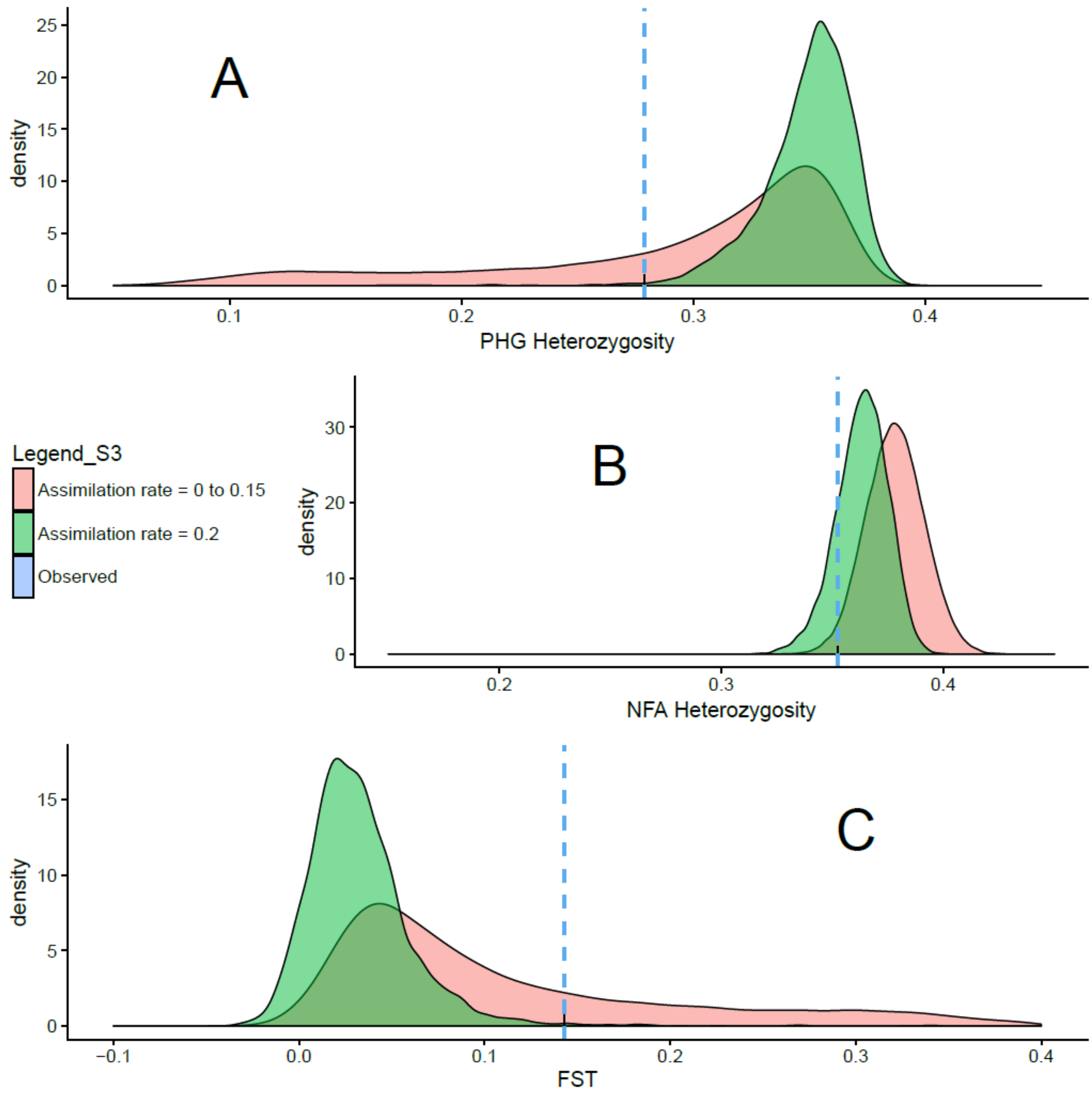

Supplement: Supplementary file 3 [file EVA-11-1642-s003.pdf]
