# Supplementary material for: Bayesian estimation of partial population continuity using ancient DNA and spatially explicit simulations
Source: Evol Appl. 2018 Jul 3;11(9):1642–55. doi: 10.1111/eva.12655 (PMC6183456; doi:10.1111/eva.12655)

Figure S4. The Quantile plots show the position of the "true" value θ in the cumulative posterior distribution for 1,000 pseudo-observed datasets, the "true" value being the parameter value used for the simulation. A) Mitochondrial γ parameter when its prior goes from 0.0 to 0.15, B) Autosomal γ parameter when its prior goes from 0.0 to 0.2, C) Autosomal γ parameter when its prior goes from 0.0 to 0.5, D) Autosomal m_{PHG} parameter in a model when γ prior goes from 0.0 to 0.2, E) Autosomal K_{PHG} parameter in a model when γ prior goes from 0.0 to 0.2.

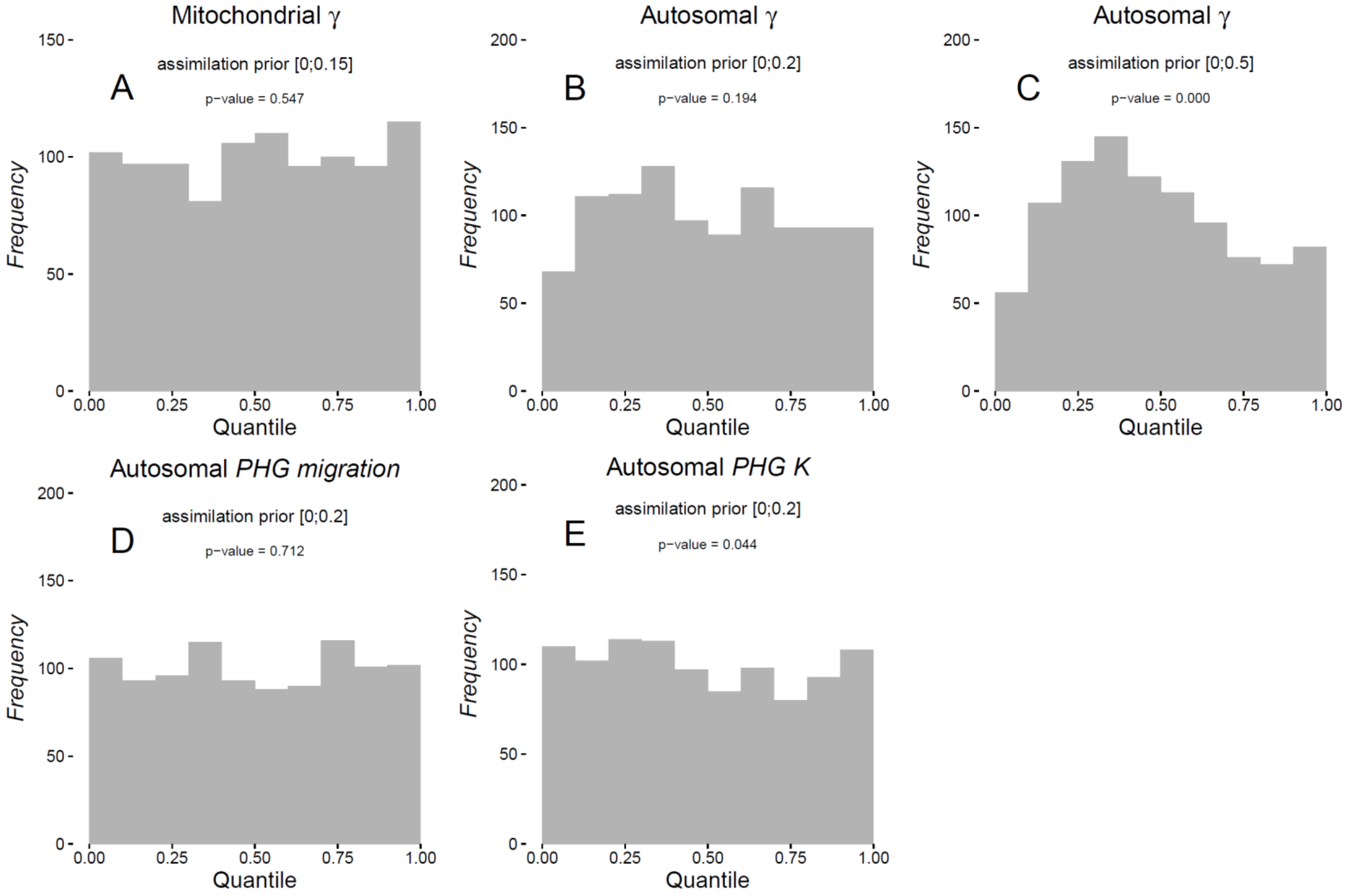

Supplement: Supplementary file 4 [file EVA-11-1642-s004.pdf]
