# Supplementary material for: Bayesian estimation of partial population continuity using ancient DNA and spatially explicit simulations
Source: Evol Appl. 2018 Jul 3;11(9):1642–55. doi: 10.1111/eva.12655 (PMC6183456; doi:10.1111/eva.12655)

Figure S5. Plots of the estimated $\hat{\gamma}$ through the mode of its posterior distribution and the "true" value γ for 1,000 pseudo-observed simulations. The green line represents the diagonal, the red line is the "lm"-type smoothed line with y ~x and the blue line is the "loess"-type smoothed curve with y ~log(x). All smoothed lines are made using the "ggplot2" R package v2.2.1. The coefficients of Spearman and Pearson are given for all cases and are statistically significant at 1% level. A) Mitochondrial dataset with γ prior distribution going from 0.0 to 0.15, B) Autosomal dataset with γ prior distribution going from 0.0 to 0.2, C) Autosomal dataset with γ prior distribution going from 0.0 to 0.5.

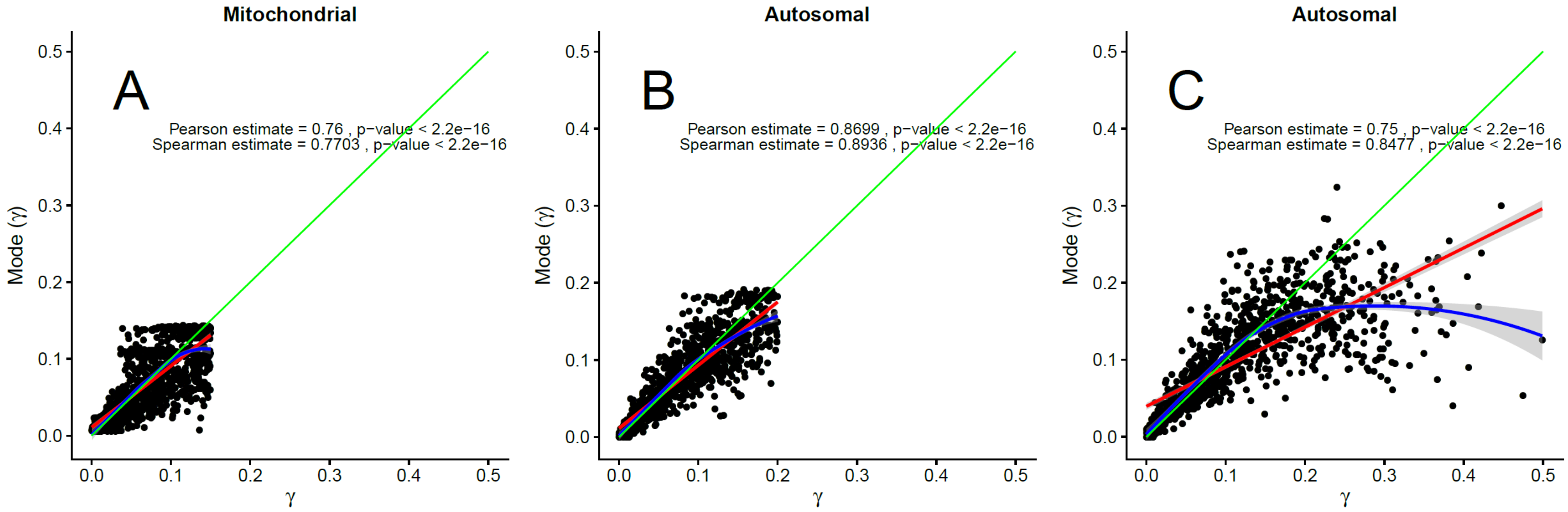

Supplement: Supplementary file 5 [file EVA-11-1642-s005.pdf]
