# Supplementary material for: Bayesian estimation of partial population continuity using ancient DNA and spatially explicit simulations
Source: Evol Appl. 2018 Jul 3;11(9):1642–55. doi: 10.1111/eva.12655 (PMC6183456; doi:10.1111/eva.12655)

Figure S6. Genetic contribution of PHG from various zones defined in Figure 3 to the NFA populations from zone A at the end of the Neolithic transition ~4,500 BP. The blue line represents the "loess"-type smoothed curve with y ~log(x) calculated with the "ggplot2" R package v2.2.1. The green line is the γ mode estimated for each DNA type and the red line is the corresponding PHG contribution in blue line coordinates A) Autosomal contribution from zone A, B) Autosomal contribution from zone A + B, C) Autosomal contribution from zone A + B + D, D) Mitochondrial contribution from zone A, E) Mitochondrial contribution from zone A + B, F) Mitochondrial contribution from zone A + B + D. The mean and standard deviation are given for each graph, as well as the mean contribution for zone A, B and D. The contribution from zone B = (A+B) – (A), the contribution from zone D = (A+B+D) – (A+B), the contribution from zone C = 1-(A+B+D).

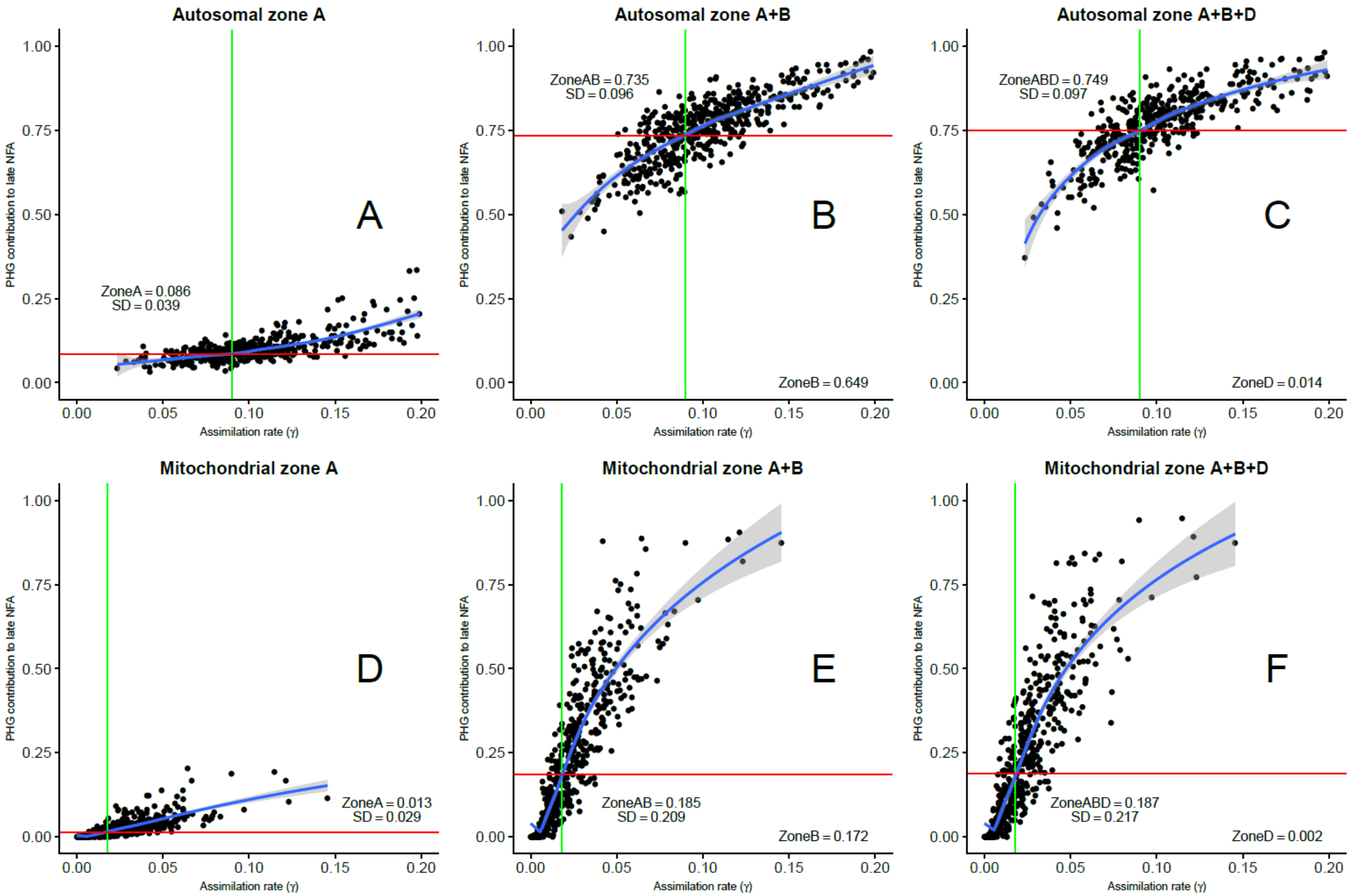

Supplement: Supplementary file 6 [file EVA-11-1642-s006.pdf]
