# Supplementary material for: Bayesian estimation of partial population continuity using ancient DNA and spatially explicit simulations
Source: Evol Appl. 2018 Jul 3;11(9):1642–55. doi: 10.1111/eva.12655 (PMC6183456; doi:10.1111/eva.12655)

Figure S7. Location on the European map of the coalescent events, when tracing back in time the lineages sampled in Central Europe at the end of the Neolithic (~4,500 BP). Blue represents water cells and white represents cells without any coalescent event. Then the different colours represent the relative frequency of coalescent events within each cell: yellow is the maximum frequency, then lighter green mean more coalescent events and darker green means less coalescent events; black is the minimum frequency. A) For one single simulation. B) For 1,000 simulations.

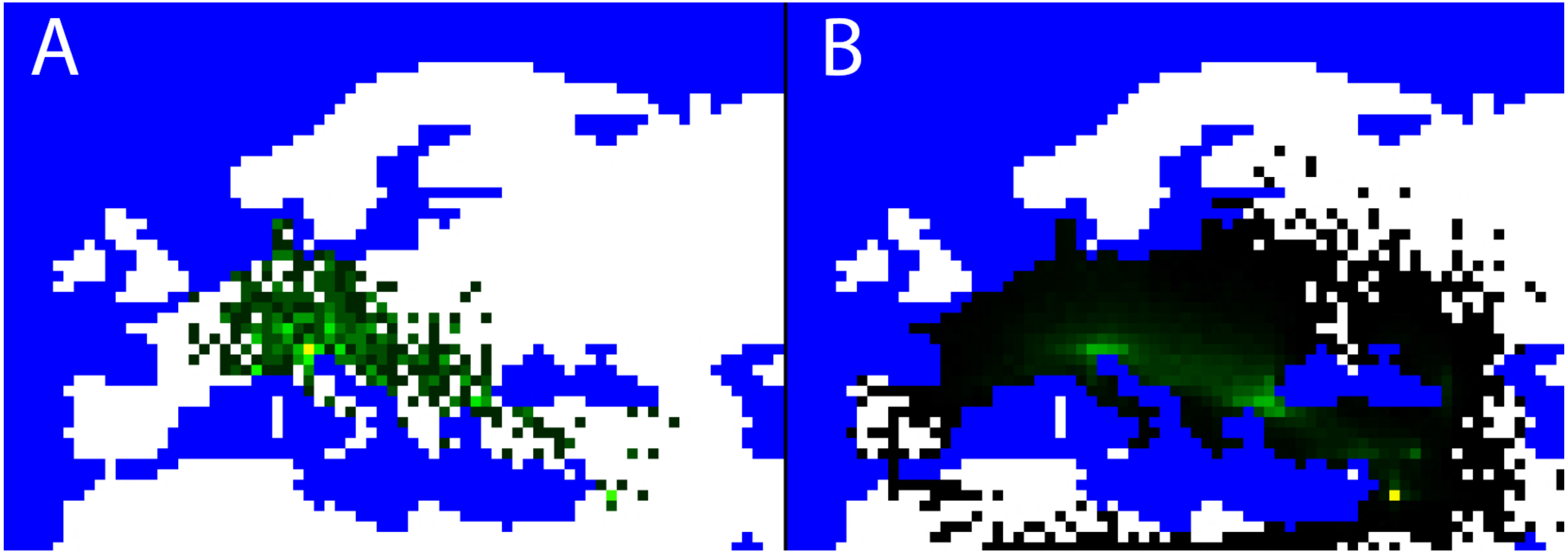

Supplement: Supplementary file 7 [file EVA-11-1642-s007.pdf]
