# Supplementary material for: Bayesian estimation of partial population continuity using ancient DNA and spatially explicit simulations
Source: Evol Appl. 2018 Jul 3;11(9):1642–55. doi: 10.1111/eva.12655 (PMC6183456; doi:10.1111/eva.12655)

Figure S8. Genetic contribution to the NFA populations from zone A at the end of the Neolithic transition ~4,500 BP, of the PHG populations located all the way of the NFA expansion from its source in the Near East. 1,000 simulations were performed with parameter values drawn from table 2, excepted γ that goes up to 0.5. The blue line is the "loess"-type smoothed curve with y ~log(x) calculated with the "ggplot2" R package v2.2.1.

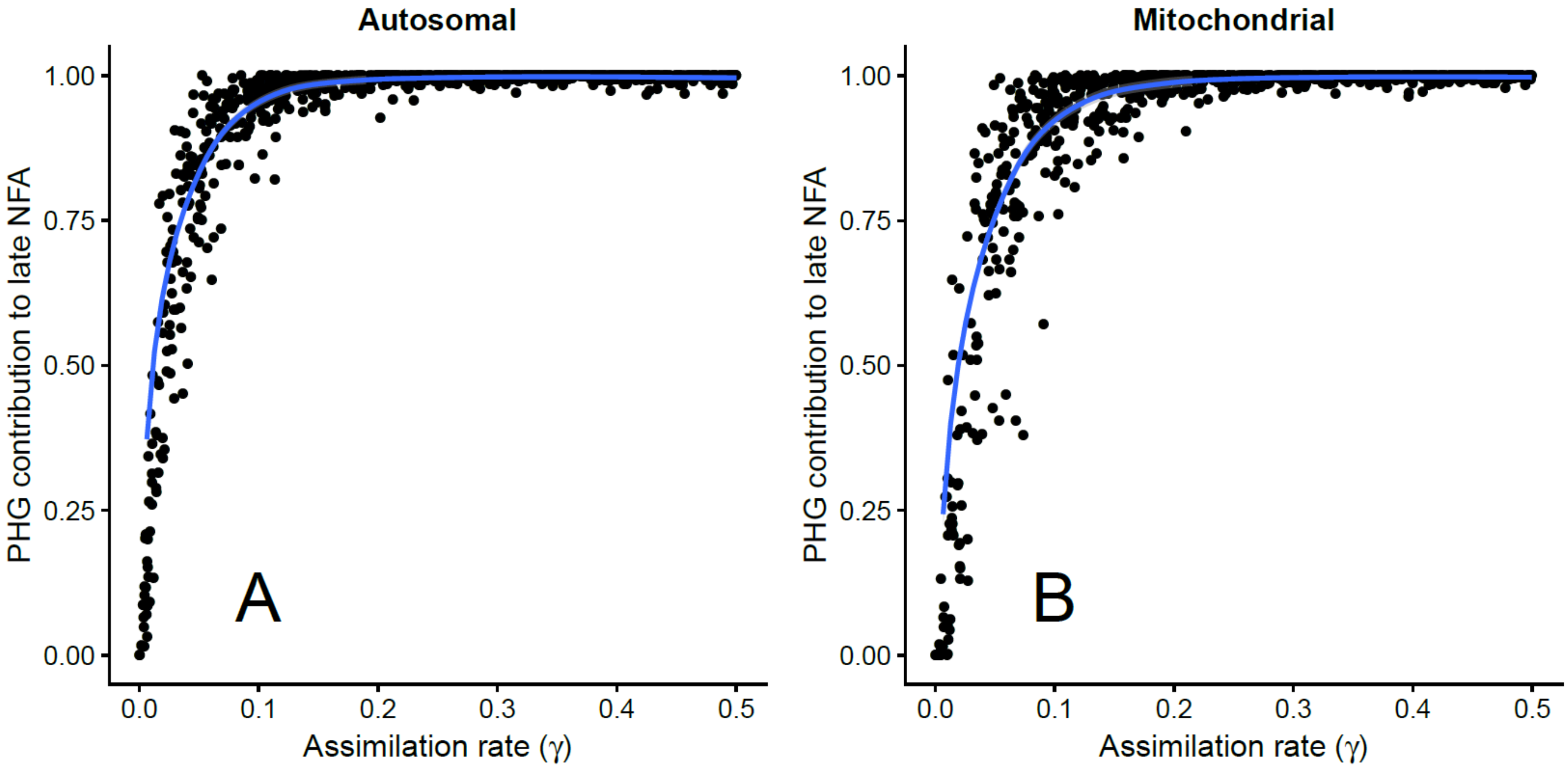

Supplement: Supplementary file 8 [file EVA-11-1642-s008.pdf]
