# Supplementary material for: Bayesian estimation of partial population continuity using ancient DNA and spatially explicit simulations
Source: Evol Appl. 2018 Jul 3;11(9):1642–55. doi: 10.1111/eva.12655 (PMC6183456; doi:10.1111/eva.12655)
Supplement: Supplementary file 9 [file EVA-11-1642-s009.pdf]

Supporting Tables S1. For each of the two datasets (mitochondrial and autosomal), 1,000 pseudo-observed simulations (pods) were generated by drawing parameters from the prior distributions of Table 2. Then γ was estimated for each pods using the procedure described in the main text for real data and 5 indices of accuracy were recorded: the relative bias = $\frac{1}{1000}\sum_{i=1}^{1000}\frac{\hat{\gamma}_i-\gamma}{\gamma}$, the relative root mean square error (RMSE) = $\frac{1}{\gamma}\sqrt{\frac{1}{1000}\sum_{i=1}^{1000}(\hat{\gamma}_i-\gamma)^2}$, the coverages 50 and 90, defined as the proportion of simulations in which the "true" value γ lies within the 50 (respectively 90) HDI around the estimate $\hat{\gamma}_i$, the factor 2 defined as the proportion of $\hat{\gamma}_i$ lying in an interval bounded by 50% and 200% of the "true" γ. The results are presented for each range of γ and the grey line indicates the range where falls the estimation (mode of assimilation rate γ) on the real data.

Mitochondrial estimation

"True" γ	Rel. Bias	Rel. RMSE	Factor2	Coverage 50	Coverage 90
0.00-0.01	2.38	< 0.001	0.16	0.58	0.98
0.01-0.02	-0.06	< 0.001	0.76	0.68	0.97
0.02-0.03	-0.06	< 0.001	0.82	0.50	0.95
0.03-0.04	0.01	0.001	0.78	0.50	0.90
0.04-0.05	0.10	0.001	0.82	0.52	0.87
0.05-0.06	0.07	0.001	0.92	0.31	0.82
0.06-0.07	0.08	0.001	0.97	0.41	0.95
0.07-0.08	0.00	0.001	0.96	0.49	0.91
0.08-0.09	-0.08	0.001	0.99	0.66	0.97
0.09-0.10	0.06	0.001	1.00	0.53	0.94
0.10-0.11	-0.06	0.001	0.94	0.58	0.92
0.11-0.12	-0.05	0.001	1.00	0.73	1.00
0.12-0.13	-0.10	0.001	0.97	0.64	0.96
0.13-0.14	-0.18	0.002	0.97	0.36	0.86
0.14-0.15	-0.23	0.002	0.96	0.36	0.80

Autosomal estimation

"True" γ	Rel. Bias	Rel. RMSE	Factor2	Coverage 50	Coverage 90
0.00-0.01	0.72	< 0.001	0.53	0.59	0.95
0.01-0.02	0.23	< 0.001	0.78	0.52	0.89
0.02-0.03	0.06	< 0.001	0.97	0.63	0.94
0.03-0.04	0.11	< 0.001	0.89	0.52	0.92
0.04-0.05	0.12	0.001	0.95	0.69	0.91
0.05-0.06	0.11	0.001	0.94	0.57	0.91
0.06-0.07	0.07	0.001	0.96	0.60	0.93
0.07-0.08	0.19	0.001	0.97	0.48	0.85
0.08-0.09	0.00	0.001	1.00	0.64	0.98
0.09-0.10	0.04	0.001	1.00	0.53	0.94
0.10-0.11	0.04	0.001	0.98	0.50	0.94
0.11-0.12	-0.01	0.001	1.00	0.61	1.00
0.12-0.13	-0.03	0.001	0.98	0.40	0.86
0.13-0.14	-0.09	0.001	1.00	0.40	0.91
0.14-0.15	-0.08	0.001	1.00	0.62	0.95
0.15-0.16	-0.09	0.001	1.00	0.60	0.91
0.16-0.17	-0.13	0.001	1.00	0.62	1.00
0.17-0.18	-0.18	0.002	0.96	0.39	0.89
0.18-0.19	-0.25	0.003	1.00	0.31	0.85
0.19-0.20	-0.29	0.004	0.91	0.13	0.78
