# Supplementary material for: Bayesian estimation of partial population continuity using ancient DNA and spatially explicit simulations
Source: Evol Appl. 2018 Jul 3;11(9):1642–55. doi: 10.1111/eva.12655 (PMC6183456; doi:10.1111/eva.12655)
Supplement: Supplementary file 10 [file EVA-11-1642-s010.pdf]

**Supporting Tables S2.**

We performed 16 simulations with fixed values of $r_{PHG}$ = 0.3, $r_{NFA}$=0.6, $K_{PHG}$=150, $K_{NFA}$=1500 and combinations of $\gamma$ (=0.0, 0.05, 0.1, 0.2), $m_{PHG}$ (=0.15, 0.3) and $m_{NFA}$ (=0.4, 0.8). $\gamma$ was chosen within its range of interest (0.0-0.2) while minimum and maximum values for $m_{PHG}$ and $m_{NFA}$ were taken from their prior distributions. The table shows the estimated $\hat{\gamma}$ and HDI 90 when using the estimation procedure described in the main text. Blue colour indicates underestimation while red colour indicates overestimation. NA stands for combinations of demographic parameter unable to sample at the dates and locations of the real samples (e.g. PHG disappear too quickly due to the competition exerted by NFA).

Mitochondrial estimation

| "True" $\gamma$ | $m_{PHG}$/ $m_{NFA}$ | | | |
|---|---|---|---|---|
| | **0.15/0.4** | **0.15/0.8** | **0.3/0.4** | **0.3/0.8** |
| **0.00** | 0.007 [0.000-0.030] | 0.01 [0.000-0.036] | 0.007 [0.000-0.029] | 0.01 [0.000-0.037] |
| **0.05** | 0.050 [0.023-0.110] | 0.060 [0.0230-0.121] | 0.047 [0.021-0.107] | 0.059 [0.031-0.122] |
| **0.10** | 0.094 [0.053-0.141] | 0.111 [0.064-0.146] | 0.092 [0.053-0.141] | 0.116 [0.068-0.146] |
| **0.20** | 0.120 [0.070-0.147] | 0.137 [0.089-0.149] | 0.121 [0.072-0.147] | 0.138 [0.091-0.149] |

Autosomal estimation

| "True" $\gamma$ | $m_{PHG}$/ $m_{NFA}$ | | | |
|---|---|---|---|---|
| | **0.15/0.4** | **0.15/0.8** | **0.3/0.4** | **0.3/0.8** |
| **0.00** | 0.000 [0.000-0.007] | 0.000 [0.000-0.008] | 0.003 [0.000-0.012] | 0.004 [0.000-0.015] |
| **0.05** | 0.046 [0.003-0.090] | 0.030 [0.0-0.066] | 0.058 [0.021-0.092] | 0.035 [0.009-0.063] |
| **0.10** | 0.135 [0.063-0.264] | NA | 0.111 [0.036-0.256] | NA |
| **0.20** | 0.229 [0.123-0.345] | NA | 0.220 [0.108-0.340] | NA |
