# Supplementary material for: Bayesian estimation of partial population continuity using ancient DNA and spatially explicit simulations
Source: Evol Appl. 2018 Jul 3;11(9):1642–55. doi: 10.1111/eva.12655 (PMC6183456; doi:10.1111/eva.12655)
Supplement: Supplementary file 11 [file EVA-11-1642-s011.pdf]

Supporting File 1 – Spatially-Explicit continuity test

Method:

Following Silva, Rio et al. (2017), we performed a test of population continuity with each of the two datasets presented in Table 1 and Figure 2 (mitochondrial and autosomal) made up of samples from pre and post Neolithic era.

In short, the test consists in simulating under the null hypothesis of population continuity, molecular samples with identical characteristics than the real ones, in terms of size, geography, chronology and molecular markers. By continuity, we mean a population evolving under the effects of genetic drift and ongoing local gene flow with neighbouring populations, without involving any large genetic input from abroad during the time frame under study. Then, the indice of genetic differentiation *Fst* is computed among samples belonging to the two time periods (pre-Neolithic hunter-gatherers and post-Neolithic farmers) using the program Arlequin (Excoffier and Lischer 2010). By repeating the simulations, it is thus possible to generate a distribution of *Fst* under the null hypothesis of population continuity. The proportion of simulation giving an *Fst* larger than the one computed from real data is thus indicative of the capability of the model to reproduce the observed data. If this proportion (*Psim>obs*) is smaller than a 5% treshold, then we consider that the observed *Fst* is sufficiently high to reject the null hypothesis of population continuity. Details about the approach are given in Silva, Rio et al. (2017).

Here we used the model described under the Materials and Methods section of the main text. Each model parameter was drawn from its prior distribution – to account for its uncertainty – excepted the assimilation rate γ which was fixed to its maximum value of 1.0. It represent the fact that the Neolithic way of life spread through cultural diffusion (Currat and Excoffier 2005) and that pre-neolithic hunter-gatherers (PHG) and Neolithic farmers (NFA) constitute a continuous panmictic population. In order to be sure to sample all ancient data (some parameters combinations with large migration rates make PHG disappear too quickly to be sampled), we modified the model by extending the period of cohabitation between PHG and NFA in zone A (Figure 3) until 4400 years BP (Bollongino, Nehlich et al. 2013).

For each of the 10,000 simulations, 10,000 SNP were generated for the autosomal dataset and sequences of 344 bp length for the mitochondrial dataset, using a mutation rate of $7.5x10^{-6}$ mutations/generation/site (Bramanti, Thomas et al. 2009).

Results:

Both dataset significantly rejected population continuity (autosomal *Psim>obs* = 0.0067 and mitochondrial *Psim>obs* =0.0037).

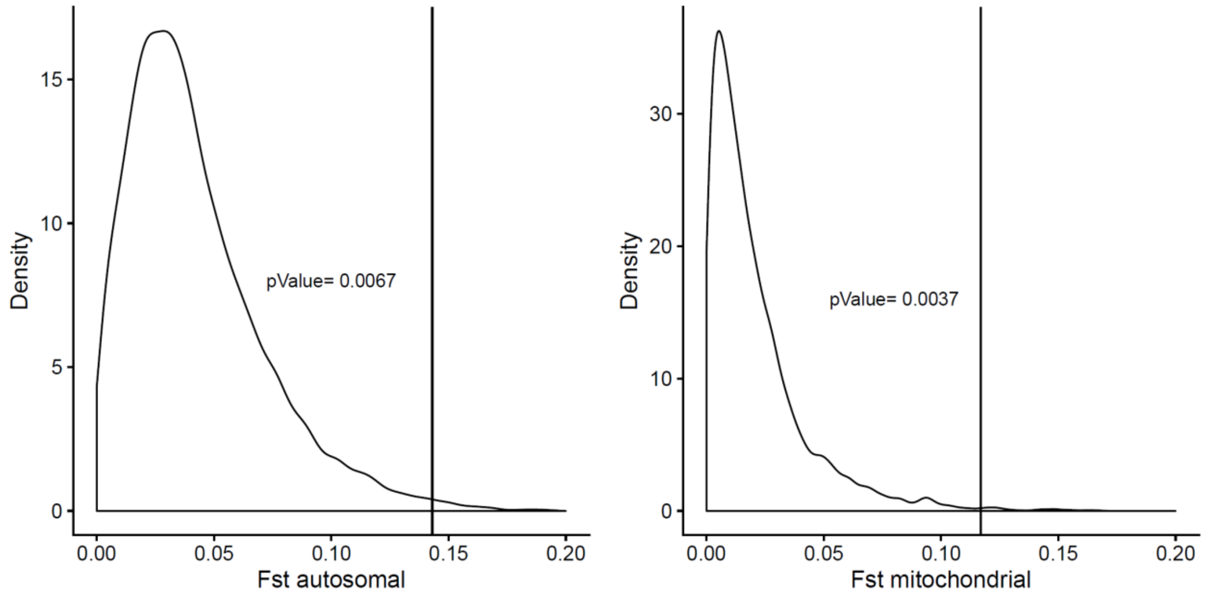

Distribution of simulated Fst under the hypothesis of population continuity. The bar shows the observed value.

References:

Bollongino, R., O. Nehlich, M. P. Richards, J. Orschiedt, M. G. Thomas, C. Sell, Z. Fajkosova, A. Powell and J. Burger (2013). "2000 years of parallel societies in Stone Age Central Europe." Science 342(6157): 479-481.

Bramanti, B., M. G. Thomas, W. Haak, M. Unterlaender, P. Jores, K. Tambets, I. Antanaitis-Jacobs, M. N. Haidle, R. Jankauskas, C. J. Kind, F. Lueth, T. Terberger, J. Hiller, S. Matsumura, P. Forster and J. Burger (2009). "Genetic discontinuity between local hunter-gatherers and central Europe's first farmers." Science 326(5949): 137-140.

Currat, M. and L. Excoffier (2005). "The effect of the Neolithic expansion on European molecular diversity." Proc Biol Sci 272(1564): 679-688.

Excoffier, L. and H. E. L. Lischer (2010). "Arlequin suite ver 3.5: a new series of programs to perform population genetics analyses under Linux and Windows." Molecular Ecology Resources 10(3): 564-567.

Silva, N. M., J. Rio and M. Currat (2017). "Investigating population continuity with ancient DNA under a spatially explicit simulation framework." Bmc Genetics 18.
